# New developments in the management of achondroplasia

**DOI:** 10.1007/s10354-020-00741-6

**Published:** 2020-03-06

**Authors:** Wolfgang Högler, Leanne M. Ward

**Affiliations:** 1 Department of Paediatrics and Adolescent Medicine, Johannes Kepler University Linz, Krankenhausstraße 26–30, 4020 Linz, Austria; 2 Institute of Metabolism and Systems Research, University of Birmingham, Birmingham, UK; 3 Departments of Paediatrics and Surgery, University of Ottawa, Ottawa, Canada; 4 Division of Endocrinology and Metabolism, The Children’s Hospital of Eastern Ontario, Ottawa, Ontario Canada

**Keywords:** Foramen magnum, C‑type natriuretic peptide, Fibroblast growth factor receptor, Growth, Spinal stenosis, Foramen magnum, C‑Typ-natriuretisches Peptid, Fibroblastenwachstumsfaktor‐Rezeptor, Wachstum, Spinalkanalstenose

## Abstract

Achondroplasia is the most common form of disproportionate short stature. A dominantly inherited *FGFR3* mutation permanently activates the fibroblast growth factor receptor 3 (FGFR3) and its downstream mitogen-activated protein kinase (MAPK) signalling pathway. This inhibits chondrocyte differentiation and puts a break on growth plate function, in addition to causing serious medical complications such as foramen magnum and spinal stenosis and upper airway narrowing. A great deal has been learned about complications and consequences of FGFR3 activation and management guidance is evolving aimed to reduce the increased mortality and morbidity in this condition, particularly deaths from spinal cord compression and sleep apnoea in infants and small children. To date, no drugs are licensed for treatment of achondroplasia. Here, we report on the various substances in the drug development pipeline which target elements in molecular disease mechanism such as FGF (fibroblast growth factor) ligands, FGFR3, MAPK signalling as well as the C‑type natriuretic peptide receptor NPR‑B (natriuretic peptide receptor B).

## Zusammenfassung

Die Achondroplasie ist die häufigste Form des disproportionierten Kleinwuchses. Eine dominant vererbte FGFR3(„fibroblast growth factor receptor“ 3)-Mutation aktiviert den FGFR3 und die downstream MAPK(„mitogen-activated protein kinase“)-Signalkaskade dauerhaft. Dies hemmt die Chondrozytendifferenzierung, bremst der Wachstumsfugenaktivität und löst zusätzlich schwerwiegende Komplikationen aus, etwa Stenosen des Foramen magnum und des Spinalkanals sowie eine Verengung der oberen Atemwege. Das zunehmende Wissen um Komplikationen der FGFR3-Aktivierung hat zur Entwicklung von Behandlungsempfehlungen geführt, die darauf abzielen, die Morbidität und Mortalität dieser Erkrankung zu reduzieren, besonders Todesfälle durch Rückenmarkkompression und Schlafapnoen bei Säuglingen und Kleinkindern. Für die Therapie der Achondroplasie ist derzeit kein Medikament zugelassen. Wir berichten über verschiedene Substanzen in der „drug development pipeline“, die auf unterschiedliche Elemente im molekularen Mechanismus der Krankheit abzielen, wie den FGF(„fibroblast growth factor“)-Liganden, den FGFR3, die MAPK-Signalkaskade und den NPR-B(„natriuretic peptide receptor B“)-Rezeptor des natriuretischen Peptids Typ C.

## Human growth plate function

The force behind human bone elongation lies in the growth plates. Prenatal and postnatal elongation of bones are mastered by these fascinating little organs that drive all mammalian long bone as well as vertebral bone growth but disappear once exposed to sufficient amounts of oestrogen at the end of male and female puberty. Despite major leaps in our understanding [1, 2], much about growth plate function remains unknown, including the various mechanisms that control them, and the reasons why some work faster than others to produce bones of different lengths and shape within and across species.

Short stature, whilst not a disease in and of itself, can affect daily life and is often met by limited societal acceptance [3], and may impact quality of life in cases of extreme short stature [4]. In clinical practice to date, recombinant human growth hormone (rhGH) and its main metabolite insulin-like growth factor 1 (rhIGF-1) are the only licensed treatments for some forms of short stature. We anticipate that this will change, since we are approaching a new drug development era for rare growth disorders that targets mechanisms other than the GH/IGF‑1 receptor pathway.

The term skeletal dysplasia refers to over 400 genetic conditions affecting bone size, shape and elongation. Apart from Turner and Prader Willi syndromes, SHOX haploinsufficiency, inherited forms of GH deficiency and primary IGF‑1 deficiency, no other genetic conditions have licenses for rhGH or rhIGF‑1 treatment. This is due to insufficient safety and efficacy data, most often related to the rarity of the particular condition, rendering clinical trials with sufficient numbers of patients challenging. Of note, the medical knowledge of genetic conditions and traits causing short stature has now expanded [5] to a level that almost makes obsolete the term “idiopathic short stature”.

## Achondroplasia—more than extreme short stature

Achondroplasia (OMIM #100800) is the most common form of disproportionate short stature, affecting 1:20,000 live births. Like some other severe growth disorders, it is also associated with potentially serious medical complications such as foramen magnum and spinal stenosis, both of which cause increased morbidity and mortality [6, 7]. Achondroplasia is caused by a heterozygous, activating mutation in the fibroblast growth factor receptor‑3 (*FGFR3*) gene at position 1138. Two specific mutations (1138G > A and 1138G > C) lead to an arginine to glycine substitution at position 380 (G380R) in the transmembrane domain of the FGFR3 protein, which permanently activates the receptor [8]. This mutated receptor, through a multistep, postreceptor cascade, puts a continuous permanent break onto chondrocyte proliferation in all growth plates. The rhizomelic appearance of individuals affected by achondroplasia suggests that faster proliferating growth plates (femur, humerus) tend to be more affected than slower ones at other sites.

Around 80% of mutations are de novo and 20% inherited, which demonstrates the reduced reproduction of affected individuals despite the dominant inheritance [6]. Fathers of de novo patients are statistically older than average fathers, which is the subject to ongoing research [9].

The achondroplasia clinical phenotype has been well-described and documented over thousands of years, consisting of a large head with characteristic facies, frontal bossing and midface hypoplasia, a long narrow trunk with exaggerated lumbar lordosis, rhizomelic shortening of the limbs, limitation of elbow extension, genu varum, and trident hands. A thoracolumbar gibbus is present in infancy, which later converts into hyperlordosis. Hyperextensibility of joints and mild–moderate muscular hypotonia lead to delayed motor milestones and worsening of hyperlordosis [10]. An extended phenotypic description has been reviewed in detail elsewhere [6].

## Spontaneous growth and body proportions in achondroplasia

Mean adult height in achondroplasia is 132 cm in males and 124 cm in females [11]. New disease-specific growth curves have recently been established, which also demonstrate that the main loss of height occurs in the first 2 years of life [11]. As expected, the early onset of disproportion is caused by reduced growth of legs and arms which worsens over time [12].

The achondroplasia mouse model recapitulates the human phenotype, including early severe growth retardation, disproportionate limb shortening, round head, mid-face hypoplasia at birth, and kyphosis progression during postnatal development. In addition, premature fusion of the cranial sutures and low bone mass were observed in newborn mice whose phenotypes became more pronounced during postnatal skeletal development [13].

## Medical complications

The increased risk of first-year deaths in infants with achondroplasia has been known since the 1980s [14, 15]. The risk of death was increased approximately 6‑fold in one study [16]. One of the factors contributing to infant mortality is foramen magnum stenosis, which can cause cervical cord compression leading to respiratory failure and sudden infant deaths.

Narrowing of upper respiratory airways due to mid-face hypoplasia can cause obstructive apnoea. The anatomic, obstructive component of breathing complicates the assessment of central breathing abnormalities caused by brain stem compression. Overall, sleep disorders (obstructive, mixed, central) affect 30–60% of all infants with achondroplasia, necessitating polysomnography screening [17]. Mid-face hypoplasia and temporal bone abnormalities also lead to chronic otitis media, which in turn can cause conductive hearing loss and speech delay, often necessitating ventilation tube insertion [6].

Overall mortality was increased in a large study of 793 individuals with achondroplasia; predominant causes of death were sudden death in children up to age 5 years and cardiovascular disease in young adults [18]. Life expectancy was reduced by 10 years. A recent study of 855 individuals also demonstrated the highest risk of death in children up to age 4 years, but with improving rates, presumably due to better assessment and intervention for brain stem compression. In subjects older than 5 years, there was an increased rate of cardiovascular, cerebrovascular and accidental deaths [19].

Spinal canal and foramen magnum stenosis originate from the same pathophysiological cause, which is premature closure of synchondroses (cartilaginous joints). Such premature closure is found both in achondroplasia and in thanatophoric dysplasia (OMIM 187600), and in achondroplasia mouse models [20]. In affected mice, chondrocyte-specific activation of Fgfr3 additionally induced osteoblast differentiation and bone formation around the prematurely closing synchondroses. The authors went on to demonstrate that high FGF signalling increased the expression of the strongly osteogenic bone morphogenetic protein (Bmp), with decreased expression of Bmp antagonists. This finding indicates a possible role of Bmp signalling in the acceleration of synchondrosis fusion, paracrine activation of osteoblast differentiation and premature unification of ossification centres. Should this be the case, then any growth-promoting treatment of achondroplasia would need to precede the timing of the synchondrosis closure in order to prevent these complications. Given the occurrence of complications from foramen magnum stenosis very early in life, the timing of future interventions would need to be shortly after, or before, birth.

Obesity is certainly an issue in individuals with achondroplasia and tends to emerge early in life. Obesity is predominantly of abdominal origin and its causes are currently not understood [21]. Approximately 50% of children are affected [22]. How obesity affects mobility, cardiovascular risk, occurrence of back pain and other complications has not been systematically studied, which supports the role of natural history studies for this rare disease.

Body mass index may not be the optimal parameter to assess obesity in patients with achondroplasia [11], due to the fact that that weight does not scale to height squared in children, which creates a size artefact in anyone who is very short [23, 24].

## From disease mechanism to drug development

Fibroblast growth factor receptors (FGFRs) belong to the tyrosine kinase family and regulate various biological processes including cell proliferation and differentiation during development, as well as tissue repair. Many genetic conditions are caused by deregulation in the FGFRs signalling network. The FGFR family consists of four family members, FGFR1–4 [25].

Mutations in *FGFR3* on chromosome 4p16.3 were first described as the cause of achondroplasia in 1994 [26, 27]. The mutation enhances the receptor’s tyrosine kinase activity and activates mainly the downstream canonical mitogen-activated protein kinase (MAPK) pathway; however, additional signalling pathways also have been implicated, e.g., STAT, Wnt/β-catenin, PI3K/AKT, and PLCγ [28].

The discovery of the molecular pathogeny of achondroplasia attracted the interest of industry in this rare disease, and strategies for drugs targeting the overactive FGFR3 receptor and downstream signalling pathways started to develop.

**Fig. 1:**
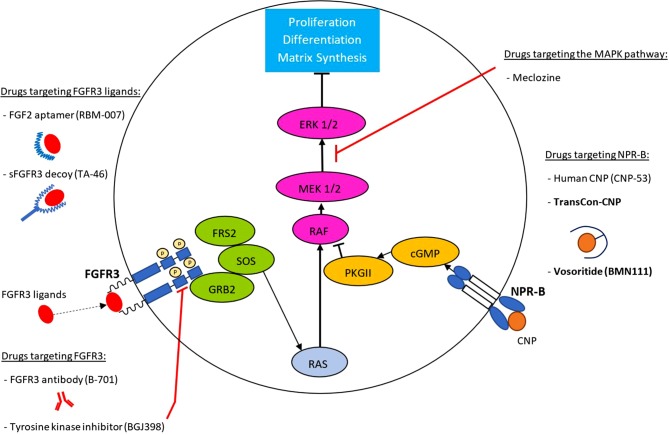
Drugs in development for the treatment of achondroplasia. Depicted is a growth plate chondrocyte. The main targets are FGFR3 ligands, the mutated FGFR3 and its activated downstream MAPK signalling pathway, as well as the NPR‑B receptor. In* bold* are substances currently in clinical trials (as of November 30, 2019). The complex MAPK pathway which originates from FGFR3, as well the MAPK-inhibitory pathway that originates from NPR‑B activation, are depicted for simplification

Current strategies include catching FGFR3 ligands, blocking FGFR3, and chemical inhibitors of tyrosine kinase, the intracellular element of the FGFR3 receptor, all of which currently remain in preclinical studies. More advanced are alternative strategies involving C‑type natriuretic peptide (CNP), which, via its receptor NPR‑B, antagonizes the FGFR3-induced activation of the MAPK signalling pathway in growth plate chondrocytes [29] and thus counteract the effects of the *FGFR3* mutation. Here we provide an overview on drug development targeting the respective pathways. Fig. 1 provides an overview over drugs in development. Whether clinical trials are being conducted was assessed on www.clinicaltrials.gov as of November 30, 2019.

## Drugs targeting the FGFR3 ligands

### Fibroblast growth factor 2 aptamer (RBM-007)

An aptamer is a short, single-stranded nucleic acid molecule that is selected in vitro to a target molecule based on its high and specific affinity. These oligonucleotides are modified to resist ribonucleases and have the ability to fold, building a three-dimensional structure that binds the target. Aptamers can be applied therapeutically due to their strong and targeted, neutralizing activities. Being an aptamer, RBM-007 (APT-F2P) is highly specific for fibroblast growth factor 2 (FGF2), one of the signalling molecules that activate the FGFR3. This RNA aptamer blocks binding of FGF2 to its four cellular receptors, inhibits FGF2-induced downstream signalling and cell proliferation, and restores osteoblast differentiation blocked by FGF2 [30]. This aptamer also inhibits the growth of FGF2-FGFR pathway-dependent lung cancer cells [31]. The drug is still in preclinical studies.

### Soluble FGFR3 decoy (TA-46)

TA-46 is a soluble, human, recombinant FGFR3 decoy (sFGFR3), which prevents FGF from binding to the mutant FGFR3. In an animal model, sFGFR3 was injected subcutaneously twice weekly to newborn Fgfr3(ach/+) mice, throughout the growth period. Effective maturation of growth plate chondrocytes was restored in bones of treated mice, growth recovered in a dose-dependent manner, and mortality decreased [32]. Treatment with TA-46 decreases abdominal obesity in this animal model [33]. TA-46 has completed phase 1 trials and has received Orphan Drug Designation from the European Medicines Agency (EMA) and the U.S. Food and Drug Administration (FDA).

## Drugs targeting the FGFR3 and downstream signalling

### Anti-FGFR3 antibody (B-701)

Vofatamab (B-701) is a human IgG1 monoclonal antibody specific targeting the FGFR3, which does not interact with other FGFRs. Since *FGFR3* mutations causes a gain-of-function of the FGFR3 receptor in a variety of cancers, B‑701 is currently in clinical trials for urothelial cell carcinoma. No preclinical studies on achondroplasia have been published. To our best knowledge, the company has discontinued development of B‑701 for achondroplasia.

### Tyrosine kinase inhibition (BGJ398)

Infigratinib (BGJ398), a tyrosine kinase inhibitor (TKI) that blocks FGFR1–3, is currently in clinical trials for bile duct and bladder cancer. In the Fgfr3Y367C/+ mouse model of achondroplasia [34] demonstrated that low doses of subcutaneously injected infigratinib reach the growth plate and have the potential to correct the achondroplasia phenotype. BGJ398 reduced FGFR3 phosphorylation and corrected the abnormal femoral growth plates and calvaria in organ cultures from mutated mouse embryos, modified growth plate organization and lead to rapid skeletal improvements including reduced intervertebral disc defects of lumbar vertebrae, loss of synchondroses, and foramen-magnum shape anomalies. BGJ398 also inhibited FGFR3 downstream signalling pathways, including MAPK, SOX9, STAT1, and PLCγ, in the growth plates of Fgfr3Y367C/+ mice and in cultured chondrocyte models of achondroplasia [34]. No clinical studies with infigratinib or other TKIs have yet been conducted in individuals with achondroplasia.

### Meclozine/Meclizine

In preclinical studies, the licensed anti-histamine and motion sickness drug, meclozine suppresses FGFR3 signalling by downregulating phosphorylation of ERK but not of MEK [35]. In low doses, this re-purposed drug demonstrates its inhibitory effect on FGFR3 signalling, thereby increasing chondrocyte proliferation and differentiation, and rescuing the short-limbed phenotype in a transgenic mouse model of achondroplasia [36]. To date, clinical studies have not been conducted.

## Drugs targeting the CNP receptor NPR-B

### CNP analogue vosoritide (BMN111)

The CNP antagonizes FGFR3 downstream signalling by inhibiting the MAPK pathway [29]. The 39-amino acid CNP (CNP-39) analogue BMN111 has an extended plasma half-life due to its resistance to neutral endopeptidase. Lorget et al. [37] demonstrated decreased phosphorylation of extracellular signal-regulated kinases 1 (ERK1) and 2 (ERK2) in achondroplasia human growth plate chondrocytes, confirming that BMN111 inhibits FGF-mediated MAPK activation. BMN111 treatment in the Fgfr3(Y367C/+) mouse model led to a significant recovery of bone growth, with an increase in axial and appendicular skeleton lengths, improvements in dwarfism-related clinical features such as flattening of the skull, reduced crossbite, straightening of tibiae and femora, and correction of the growth plate defect. The authors concluded that their results provided proof of concept that BMN 111 might benefit individuals with achondroplasia and hypochondroplasia [37].

In 2019, the results of a phase 2 dose-finding and extension study (NCT02055157 and NCT02724228) using BMN111 (vosoritide) in 35 children with achondroplasia (aged 5–14 years) were reported [38]. The drug was given as a once daily subcutaneous injection and a dose of 15 mcg/kg was established. The first 6 months of treatment demonstrated a dose-dependent increase in the annualized growth velocity, and a sustained increase in annualized growth velocity of 1.5 cm/year was observed for up to 42 months. The most common adverse events were injection-site reactions. Serious adverse events occurred in four patients, including obstructive sleep apnoea, tonsillar hypertrophy, thyroglossal cyst, and syrinx. Therapy was discontinued in 6 patients.

### TransCon CNP

TransCon CNP is a pro-drug, consisting of CNP (CNP-38) conjugated via a cleavable linker to a polyethylene glycol carrier molecule. The pro-drug is injected once weekly subcutaneously and slowly releases active CNP to provide sustained systemic CNP levels. Preclinical data in mice and cynomolgus monkeys have shown efficacy of CNP, which avoids high systemic CNP bolus concentrations which can induce cardiovascular side effects [39]. A phase 2 clinical trial in children commences in 2020 (NCT04085523).

### Human CNP (CNP-53)

Another CNP peptide in development is the human CNP with 53 amino acids (CNP-53) which has been tested in CNP-KO rats which are phenotypically similar to CNP-KO and FGFR3-KO mice. After subcutaneous administration of human CNP-53 from 5 weeks of age for 4 weeks, the impaired longitudinal skull length, craniofacial morphology and foramen magnum size improved at 9 and 33 weeks of age, indicating at least partial rescue. Whilst synchondrosis at the cranial base in CNP-KO rats normally closes at 9 weeks, this closure was incomplete in CNP-KO rats treated with CNP-53. Since skeletal findings in CNP-KO rats resemble human achondroplasia, treatment with CNP-53 or a CNP analogue may restore craniofacial morphology, foramen magnum size and short stature [40].

## Other debated approaches—past and present

In contrast to increasing NPR‑B stimulation through providing more CNP or more potent NPR‑B ligands, limiting CNP clearance is an alternative way to ensure NPR‑B activation. CNP is cleared by the NPR‑C receptor and blocking that receptor increases circulating CNP. A recent study used a transgenic mouse overexpressing osteonecrin, a NPR‑C ligand without natriuretic activity, and successfully demonstrated increased bone growth in these mice [41]. No report has been published on an achondroplasia mouse model.

In 2012, based on decreased expression of parathyroid hormone related peptide in growth plate chondrocytes of an achondroplasia mouse model, intermittent injections with teriparatide (PTH1-34) were used which increased naso-anal length, limb growth and delayed synchondrosis closure [42]. No further studies have been published since.

In 2014, intraperitoneally injected statins rescued long bone growth in a mouse model of achondroplasia Fgfr3^Ach^ [43] but a later study demonstrated that statins do not inhibit FGFR3 signalling in chondrocytes [44].

Growth hormone is licensed for the treatment of achondroplasia only in Japan, with varying height gains reported between 2.8–4.2 cm in females and 3.5–8 cm in males. However, evidence is very limited and no randomized controlled trials have been conducted [45].

## Future perspective

As we embark on a new era of drug development in achondroplasia, a number of considerations merit particular attention. One of the foremost is that in any rare disease, the success of novel drug development is intimately linked to an in-depth knowledge of the condition’s natural history. Without natural history data, imprecision about optimal timing of drug initiation for best results, lack of knowledge about disease prevalence for sample size determinations, side effects erroneously attributed to drug instead of disease-related complications (or vice versa), and lack of understanding about best outcome measures can thwart efforts in clinical trials. To this end, long-term natural history and registry-type studies should ideally precede, or be conducted in parallel with, clinical drug trials, assessing the clinical outcomes of most relevance to the patient, and with rigorous safety data capture.

In addition, clinical drug trials are best developed on a backbone of appropriate and comprehensive standards of care. Multidisciplinary clinics are needed to address the complex and serious needs of this patient population, grounded in state-of-the-art care pathways that include both pharmacologic and nonpharmacologic intervention. Expertise in the management of foramen magnum and spinal stenosis are particularly needed, along with obstructive apnoea in infants. Management guidance that reduces infant mortality and morbidity [6, 17, 46–48] needs further development.

Given the vital role of FGF signalling in widespread cellular functions, drug specificity for the FGFR3-mediated achondroplasia pathway is essential to minimize serious side effects. It remains to be seen, for example, whether agents such as pan-specific FGFR TKIs show sufficient results in preclinical toxicology studies to undergo testing in achondroplasia. The fact that short stature syndromes by definition necessitate intervention in the young, prior to epiphyseal closure, underscores the importance of understanding the full effects of novel drugs given to the physically immature. Finally, the path that is forged through pursuit of novel therapies for achondroplasia will hopefully shed light on other growth disorders, in particular hypochondroplasia.
